# Construction and validation of a logical model for specialized Rehabilitation Centers

**DOI:** 10.11606/s1518-8787.2021055002976

**Published:** 2021-09-21

**Authors:** Regis de Souza Valentim, Thaissa Hamana de Macedo Dantas, Flavia Christiane de Azevedo Machado, Camilla Medeiros Araújo, Marilene Soares da Silva, Luciana Castaneda, Diego Dantas

**Affiliations:** I Universidade Federal do Rio Grande do Norte Faculdade de Ciências da Saúde do Trairi Programa de Pós-Graduação em Saúde Coletiva Santa CruzRN Brasil Universidade Federal do Rio Grande do Norte. Faculdade de Ciências da Saúde do Trairi. Programa de Pós-Graduação em Saúde Coletiva. Santa Cruz, RN, Brasil; II Universidade Federal do Rio Grande do Norte Departamento de Odontologia Programa de Pós-Graduação em Saúde Coletiva NatalRN Brasil Universidade Federal do Rio Grande do Norte. Departamento de Odontologia. Programa de Pós-Graduação em Saúde Coletiva. Natal, RN, Brasil; III Universidade Federal do Rio Grande do Norte Departamento de Saúde Coletiva NatalRN Brasil Universidade Federal do Rio Grande do Norte. Departamento de Saúde Coletiva. Natal, RN, Brasil; IV Universidade Federal de Pernambuco Departamento de Fisioterapia Programa de Pós-Graduação em Fisioterapia RecifePE Brasil Universidade Federal de Pernambuco. Departamento de Fisioterapia. Programa de Pós-Graduação em Fisioterapia. Recife, PE, Brasil; V Secretaria Estadual de Saúde do Rio Grande do Norte NatalRN Brasil Secretaria Estadual de Saúde do Rio Grande do Norte, Natal, RN, Brasil; VI Instituto Federal de Educação, Ciência e Tecnologia do Rio de Janeiro Rio de janeiroRJ Brasil Instituto Federal de Educação, Ciência e Tecnologia do Rio de Janeiro, Rio de janeiro, RJ, Brasil

**Keywords:** Rehabilitation centers, organization & administration, People with disabilities, rehabilitation, Resource Management of the Health Care Team, Evaluation of Processes and Results in Health Care, Human Resources Administration

## Abstract

**OBJECTIVE:**

To build and validate a logical model for health care in Specialized Rehabilitation Centers (CER) by analyzing the work process and organizational issues of centers in Rio Grande do Norte.

**METHODS:**

This is a methodological study developed in three stages: 1) documentary research of legislation and ordinances concerning the healthcare service and the Disability Care Network (RCPD); 2) focus groups with a Census study of the CER in Rio Grande do Norte to understand and assess the daily activities of the service; and 3) systematization of the information collected and, finally, proposition and validation of the evaluative logical model.

**RESULTS:**

The model encompassed five central categories of the work and organizational process: “demands”, “resources” (inputs, financial and workforce), “processes”, “products and results” and “mission, values and external factors”.

**CONCLUSION:**

The logical model built was suitable for graphical representation of the work process and organizational issues of the SRC. The study showed that the functioning of the services is in line with the regulations. However, there are still organizational gaps that need to be addressed to improve the resolution capacity of the service and the articulation with other points of the network.

## INTRODUCTION

Due to the greater number of people living with disabilities, increased incidence of chronic diseases and population aging, the subject of rehabilitation has gained prominence in Brazil and the world^1^. The agenda “Rehabilitation 2030: a call for action”, proposed by the World Health Organization (WHO), advocates universal access to rehabilitation and calls on nations to reflect on how they set up public policies, structure their assistance services and information systems^1,2^. In Brazil, the scarce literature and instruction regarding rehabilitation services, offered by Specialized Rehabilitation Centers (CER), indicates the urgency and relevance of studies that evaluate this area of care.

According to the WHO, one in seven people in the world lives with disabilities^3^. Brazilian data are out of date, since the most current overview is provided by the 2010 Census. According to this survey, 23.9% of the Brazilian population has at least one type of disability, and prevalence increases with age^4^.

Created by Ordinance No. 793/2012, which established the Disability Care Network (RCPD), the CER are health services providing specialized rehabilitation care for people with disabilities throughout the national territory^5^. These are reference centers for RCPD and for Brazilian public policies for disability care^6^.

Specialized Rehabilitation Centers can assist people with physical, hearing, visual, intellectual and multiple disabilities. According to the epidemiological profile of the territory, these centers can be authorized in three formats: CER II, comprising two types of rehabilitation; CER III, comprising three types of rehabilitation; and CER IV, comprising four types of rehabilitation (physical, visual, hearing and intellectual), in addition to the orthopedics workshop^5^.

Among the aspects of health evaluation, the analysis of implementation of programs or services through logical models has been gaining prominence^7^. The logical model is a graphical representation that shows the various possible relationships between the planned activities and the expected results^8^, making it easier for the service to plan actions and for the subjects involved therein to communicate^7^. In this perspective, the logical model is a useful tool to develop, implement and evaluate complex processes, as it allows an overview that can identify key activities and outcomes^8,9^.

Therefore, considering that the construction of these models for rehabilitation services provides an overview of the complex processes involved, generating relevant information for decision-making, this paper aims to build and validate a logical model for specialized care in CER. To achieve this, the research begins with the analysis of the work process and organizational issues in Rio Grande do Norte State.

## METHODS

This is a descriptive methodological study, with a qualitative approach, developed in three stages: documentary analysis, focus groups and, finally, construction and validation of a logical model representing the functioning of CER^8,10^. The research protocol was approved by the Research Ethics Committee of *Faculdade de Ciências da Saúde* do Trairi under CAAE no. 07082819.3.0000.5568. The study was conducted in accordance with the Helsinki Declaration, and the participants signed an informed consent form and authorized their voices to be recorded.

The first stage consisted in the study of ordinances, legislation and documents concerning the RCPD and the CER. The documentary analysis allowed us to understand the guidelines and standards applicable to the Health Network and the service that was to be evaluated. It also allowed the prior identification of key concepts useful for building the logical model^10^.

In the second stage, focus groups were organized with workers and managers to understand daily processes of the service^11^. To optimize logistics issues while maintaining sample representativeness^12^, we opted for a census sampling, with all CER from one state RCPD. For the convenience and insertion of researchers, nine CER from Rio Grande do Norte were chosen, which combined represent the state’s eight health regions. Each service was represented by a manager and a care professional, thus forming an equal composition. Professionals from different classes participated in the focus group: social service, nursing, physiotherapy, speech therapy, medicine, dentistry, psychology and occupational therapy.

The focus group sessions took place monthly as part of the RCPD Management Forum. Initially, only the manager of each service had been invited. However, at the request of the services themselves, workers who already regularly participated in the network management forums were included. In addition to the two service representatives (the manager and a care professional), two health professionals from outside the RCPD, with experience in health management and evaluation, took part in the focus groups.

The group was conducted by a team comprising a moderator and two observers, who took note of aspects concerning the non-verbal language of the participants (identified with badges) and the order and content of their testimonies^11^. Throughout the focus groups, we sought to understand the following aspects in the participants’ testimonies: service demands, causes and consequences of demand, service objectives, target audience, resources, actions, products, results and factors that interfere with service performance^10^.

In all, five meetings were held between March and July 2019, each lasting an average 90 minutes. The audio was recorded with two digital recorders positioned at the ends of the tables. The testimonies were later transcribed, and the information categorized by content theme analysis^11^, based on the theoretical framework of disability care, network care and health assessment.

The logical model was developed by the researchers based on the information collected in the previous steps, but without the presence of professionals associated with the CER^10^. For graphic presentation, an adapted version was built based on a proposal by Tamaki et al.^13^. The domains “mission”, “values” and “external factors” were added to the model.

The model was submitted to content validation in the last focus group with the CER representatives^10^. At this meeting, the group moderators presented the logical model in a multimedia projection and explained the domains included to the participants: demands, resources (inputs, financial resources and workforce), processes, products, results and external factors. The team then checked items and ideas contained in the model, domain by domain. Health service representatives expressed their opinions freely and suggested exclusions, inclusions or modifications to the allocation of items. The final version, to be considered validated, should be approved by consensus among the participants.

## RESULTS

Created in 2012 as the main component of specialized care for people with disabilities, the CER are responsible for delivering rehabilitation and habilitation actions. These centers coexist with single-type rehabilitation services (mostly philanthropic), seeking to fill the care gap and promote comprehensive disability care in articulation way with other points of the RCPD^14^.

Specialized Rehabilitation Centers are part of a public health policy comprising pre-established agents, structures, processes and care goals^15^. This policy, guided toward functioning and the biopsychosocial model, aims to offer rehabilitation services consistent with the principles and guidelines of the RCPD.

This policy is still being implemented, and it can be said that the mission of the CER is still poorly understood by both workers and users of the health system. In the day to day of the services, there are obvious difficulties regarding coverage, access, quality of care, therapeutic planning, human and material resources.

Considering that certain organizational work arrangements may prevent the comprehensiveness of care^16^, the aim was to develop a logical model of intervention that would answer the following questions: How do CER work? What are the elements of the Brazilian policy for disability care and how do they relate to one another? Do the objectives proposed in this policy move towards inclusion? What are the reasons that lead the rehabilitation policy to follow, or not, the RCPD guidelines?

The logical model lists the main topics of the organizational and working processes of CER, dividing them into six domains (Figure 1). It is worth noting that the model built from theoretical framework and discussions in the focus groups was approved by consensus, without modifications. Box 1 summarizes the themes and representative elements of the domain covered in the model.

**Figure:**
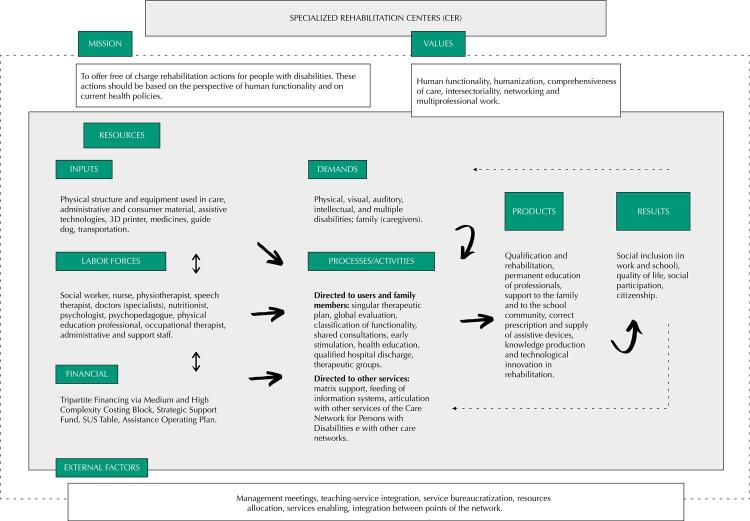
Logical model for specialized Rehabilitation Centers.

**Box 1:** Detailing of the constituent elements of the model.

Domain and definition^a^	Main comments
**Demands** The subjects and their problems.	Disabilities can be acquired throughout life, and every subject is a potential user of Specialized Rehabilitation Centers (CER). The needs of families and caregivers should be included as service demands, which expands the target audience of CER actions.
**Resources** Resources can be financial, labor or inputs.	Tripartite financing by the medium and high complexity costing Bloc for Medium and High Complexity Funding (MAC). This type of financing can be a major obstacle to the allocation of resources in the recipient service, which is not guaranteed by these administrative proceedings. Incompatibility of the values offered by the Management System of Table of Procedures, Medications, Orthotics and Auxiliary Means of Locomotion, (Sigtap) and the market values. The human resources are the multiprofessional team, composed of different healthcare categories, in addition to the support and administrative teams. The difficulty of professionals to work in an interdisciplinary manner, delivering comprehensive care focused on human functioning is noticeable. This difficulty seems to be due to how the work is formated and to the qualifications of the professionals, since CER are a recent service.
**Processes** Actions and tools to achieve the expected products and results.	Global assessment, with classification of human functioning through the International Classification of Functioning, Disability and Health – although this classification is a reality in few services. Shared consultations, unique therapeutic project, health education actions, therapeutic groups and qualified hospital discharge. Assistance actions, matrix support, feeding of information systems and articulation with other services.
**Products** Materialization of the effort expended in the processes.	Providing habilitation and rehabilitation for people with disabilities, helping these subjects to perform their daily functions and activities independently, in addition to ensuring family support capable of ensuring a harmonious coexistence between the person with disabilities and their nuclear family.
**Results** Repercurssion and medium and long term impact.	Promoting social inclusion of persons with disabilities, whether at school or work environments, improving their quality of life and ensuring social participation and the exercise of citizenship.
**External factors** Factors that are outside the scope of teams and can interfere with the performance of the service.	Factors that impact positively: the Forum for Management of the Care Network for Persons with Disabilities, a space for consultation and deliberation on the services delivered, which enables the exchange of experiences and mutual help between services, promoting quality care; partnerships between CER and technical and higher education institutions to qualify human resources and allow training workers to experience the service in practice. Factors that impact negatively: inflexibility of the ordinances governing the CER; difficulty with allocation of resources, intended exclusively for covering costs; lack of instruments to assess the quality of services and actions; difficulty in performing counter-referral and referral; manner of authorization of services by types of disability, disregarding the comprehensiveness of care and limiting the individual’s rehabilitation process.

^a^ The domains of the logical model were defined according to the theoretical model proposed by Tamaki et al.^13^

During the focus groups, we sought to identify real difficulties and problems faced daily by managers and works, as well as causes, consequences and solutions to these problems. A summary of this information is given in Box 2.

**Box 2:** Summary of the problems and challenges experienced in the routine of the service as reported by workers and managers.

Problems	Causes	Consequences	Coping strategy
Low visibility of people with disabilities.	Social stigma, lack of information about disabilities.	Social exclusion and isolation. Undervaluation of services and disability care (PcD).	Educational actions on the rights of Persons with Disability and the importance of Specialized Rehabilitation Centers (CER).
Difficulty to apply the biopsychosocial model on the overall assessment, when setting therapeutic goals and on qualified hospital discharge.	Communication deficit between workers and between workers and patients/families. Biology-centered practices and fragmented assessment by different workers. Low or no adherence to the International Classification of Functioning, Disability and Health (ICF) or instruments based on it. Resistance of families / caregivers to accept discharge, due to fears regarding the continuity of care in a home environment or continuity of disability welfare payments (BPC). Low capacity of workers to manage care and lack of evaluation of the quality of care.	Fragmentation of care and therapeutic projects based on the biomedical model, with unilateral practices established without dialogue. Difficulty to set therapeutic priorities and allocate professionals to cases. Lack of autonomy of patient and families in to manage care. Low resolution of actions, with patients staying longer than necessary and increased repressed demand.	Periodic meetings between workers and family members to develop a unique therapeutic project. Global assessment based on the ICF, with the presence of the entire team of workers. Articulation with other points of the network for continued care. Construction and revision of protocols to guide health car delivered by the CER. Development of indicators for evaluation and monitoring of the quality of care delivered in CER.
Difficulty to articulate the CER with other services of the disability care network with other care networks and with other sectors.	Little recognition and appreciation of CER within care networks. Communication failure between the CER and other RCPD services and other care networks. Low qualification of health workers and other sectors to deliver care for people with disabilities and promote inclusion. Logistical difficulties such as lack of sanitary transport and compatibility of schedules.	Little or no intersectoral articulation. Discontinuity of care and social inclusion of persons with disabilities. Worsening distance between specialized attention, other points of the network and social participation of persons with disability.	Periodic meetings with the presence of representatives of attention networks and sectors with other levels of technological density. Development of flowcharts to illustrate the relationship between services and protocols for referral.
Inflexibility of ordinances of the Ministry of Health and allocation of resources.	Disregard of local peculiarities and realities. Poor or non-existent communication between the CER and the Ministry of Health. Incompatibility of the values in the SUS table with the actual cost of products / services. Monthly resource for funding. Mismanagement of resources. Joint resources for CER and other services. Bureaucratization when implementing new CER and lack of interest of managers in doing so.	Fragmentation of care by type of disability, causing patients to travel to several points of the network in cases of multiple disabilities or different habilitation needs of the nearest CER. Need to reallocate other resources to meet the financial demands or diversion of CER resources allocated by MAC. Difficulty regarding technological advancement of the service. High demand for the service and assistance delivered not adequate to the reality of the territory.	Publication of studies on CER to problematize the need for changes in normative instruments. Listening to agents involved with CER to develop guidelines applicable to the service and attention to persons with disability. Reconsidering qualifications by type of disability and creating regulatory systems for access to specialized care and therapeutic inputs such as orthoses and prostheses.
Difficulties concerning Information Systems	Use of the information in the systems for transfer of funds. Electronic medical records not unified between primary care and CER. Information systems based on the diagnosis and biological factors of disability, disregarding the influence of context in the production of disabilities.	Information systems do not reflect the quality of services, which end up being evaluated poorly. Fragmentation of the individual’s health information, hindering access to data by rehabilitation teams and Family Health Strategy, which compromises the comprehensiveness of care. Lack of information systems that allow issuing data on functioning, limitation to activities, restriction to participation and contextual factors.	Encouraging an organizational culture that promotes values of cooperation in the service, planning of actions and evaluation of such actions. Expansion of the electronic medical record of citizens in the e-SUS to include information about CER, enabling a general analysis of individuals through their medical records. Use of information systems with ICF-based data.
Difficulties related to the permanence and qualification of workers, adequate physical structure and alternative therapies.	Workers bound to the service by contract. Low affinity of workers with the CER care model. Back-to-back work shifts, with activities in other care delivery places. Insufficient financial resources for continued education of teams.	High turnover of the care teams. Difficulty in establishing and maintaining the professional-patient bond. Team qualifications are out of date. Curative treatment, based on the biomedical model.	Public tenders and performance assessment to encourage the permanence of workers fit for the service. Periodic evaluation of CER to check on issues related to structure, processes and results obtained.

## DISCUSSION

The logical model is an important tool for decision making in health care because it allows a team to have an overview of the processes developed, whereby it can identify strengths and obstacles to obtaining the expected outcomes (whether products or results)^8^. The model proposed here, based on a case study of services that comprise RCPD in Rio Grande do Norte, has been validated by professionals and is in line with international guidelines for care management and rehabilitation^17^ and with CER standardization in Brazil^5,21,22^.

In comparison with other countries, the creation of policies and programs aimed at people with disabilities is still recent in Brazil. The National Health Policy for Persons with Disabilities^21^ and Ordinance No. 793/2012 are milestones in this process^5^. This ordinance sets the tasks of CER: to provide proper diagnosis and treatment in a timely manner; to grant, adapt and maintain assistance technologies; to act in an articulated manner and as a matrix on the subject of disability with other points of the network; and to prevent deficiencies and further aggravation^5^.

The services offered by the CER strengthen the rights won by people with disabilities over the years^21^. These centers, as the logical model presented here shows, are in line with WHO recommendations to strengthen rehabilitation in health systems. The CER ensure community access to rehabilitation services, providing a multi-professional force and allocating financial resources to offer assistance devices and technologies to all who need them. They also provide adequate training on how to use them safely and effectively^22^. However, it is noteworthy that greater integration is needed between the different levels of care and services, taking into account the dynamic limitations and needs of individuals who, in addition to health, may require education, work, leisure and culture, among others^22,24,25^.

The CER are recent services within the logic of the SUS^23^, and it became clear in the focus group that some actions have not been consolidated yet. As an example, difficulties regarding global assessment and use of the International Classification of Functioning, Disability and Health (ICF) in therapeutic planning can be cited. Professionals also highlight the lack of social assistance, quality indicators and qualified hospital discharge indicators with timely referral to other services.

The ICF should be regarded as one of the pillars of the philosophy of disability care^5,22^. Its use in therapeutic planning allows producing data and feed information systems in a standardized way. With ICF, it is also possible to optimize the allocation of professionals and identify the necessary interventions for each case, directing the therapeutic process to human functioning, ceasing to consider solely the biological function that is temporarily or permanently impaired^1,20,22,26^. Thus, the use of ICF shifts therapeutic planning from a biomedical perspective to a biopsychosocial and holistic model, contributing to a better design of the services and the development of public policies aimed at people with disabilities^2,20,26^.

As for discharge, the focus group participants considered it a problematic moment in the CER routine. To reverse this scenario, disability care needs to articulate different services and levels of attention^27^. Specialized Rehabilitation Centers need to be seen as places for specialized attention, and not as the only service for people with disabilities. They must function as the matrix^23^ to coordinate care actions and raise awareness of society and other services about their role in society, in order to facilitate the understanding of those involved in care and to optimize access and referrals. When this does not happen, communication channels between workers and services become inefficient, and failure to understand the role of CER within the network can result in underutilization of their actions^27,28^.

The lack of an evaluation culture permeates the Brazilian health system^29^. Besides the lack of internal evaluation of the quality of processes and services delivered, there is a lack of professionals working in rehabilitation. The uneven distribution of health workers and services aggravate the situation of assistance to people with disabilities, which has shown quality below ideal^29,30^.

Among the external factors interfering with the performance of the service, criticism of the way CER are is a central issue. When referral services are authorized for only some types of disability (albeit based on territorial diagnosis), attention becomes fragmented as it disregards various disabilities. The health needs of people are not standardized and can vary within the same health territory, so that it would be necessary to relax regulations for each territory to adapt the service to its reality and, at the same time, ensure access to rehabilitation services in a timely manner, without making patients have to travel long distances^22,29^.

In addition, specialized care services are concentrated in capitals, metropolises and regional centers^23^. This concentration forces people with disabilities to travel long distances to seek care, which goes against the WHO recommendation that rehabilitation services should be available as close to communities as possible, including rural communities^23,26,30^.

Although based on a study conducted in only one state of the Brazilian federation, the model presented here was shown to be in line with national and international theories and standards. Nevertheless, its use in services in other regions should consider the context of each service. In addition, future studies should include the perceptions of users in addititon to the perceptions of workers and managers. This approach can promote the protagonism of people with disabilities, as recommended by the International Convention on the Rights of Persons with Disabilities^23^.

A limitation of this study was the lack of care professionals in some focus groups due to schedule conflicts and other appointments. However, we believe that this limitation did not have a significant impact on the results of the study, since the model was validated without corrections. In addition, all participants were indicated by the services themselves and had previous experience with CER.

By comparing the validated model - which represents the ideal functioning of the services - with the local reality, workers and managers will be able to evaluate the services in which they operate, identifying potentialities and challenges. The logical model can thus work as a management tool for knowing the implementation of the service, for aiding decision-making in health care and, ultimately, favoring the planning of care actions for people with disabilities.

In addition, having identified the crucial points of the service, the model can be useful for setting up performance indicators applicable to CER, thus helping in the development of an evaluation culture in the services, either by internal or external processes of quality evaluation.

The logical model and the identification of the problems associated with the organizational structure of the CER show that the rehabilitation policy for people with disabilities is still being implemented in Brazil. Although such a policy is designed to be in line with the most up-to-date care guidelines, in reality there are obstacles in the access, patient inflow and the actions provided. It is therefore necessary to reorient the service toward complying with the guidelines of comprehensive care, focusing on human functioning, and achieving the expected results: improving quality of life and promoting social inclusion of people with disabilities.
