# Supplementary material for: Construction and validation of a logical model for specialized Rehabilitation Centers
Source: Rev Saude Publica. 2021 Sep 21;55:54. doi: 10.11606/s1518-8787.2021055002976 (PMC8455145; doi:10.11606/s1518-8787.2021055002976)
Supplement: Translation

# Translation: Construção e validação de modelo lógico para Centros Especializados em Reabilitação

**Authors:** Regis de Souza Valentim, Thaissa Hamana de Macedo Dantas, Flavia Christiane de Azevedo Machado, Camilla Medeiros Araújo, Marilene Soares da Silva, Luciana Castaneda, Diego Dantas

**Affiliations:** I Brasil Universidade Federal do Rio Grande do Norte. Faculdade de Ciências da Saúde do Trairi. Programa de Pós-Graduação em Saúde Coletiva. Santa Cruz, RN, Brasil; II Universidade Federal do Rio Grande do Norte. Departamento de Odontologia. Programa de Pós-Graduação em Saúde Coletiva. Natal, RN, Brasil; III Brasil Universidade Federal do Rio Grande do Norte. Departamento de Saúde Coletiva. Natal, RN, Brasil; IV Brasil Universidade Federal de Pernambuco. Departamento de Fisioterapia. Programa de Pós-Graduação em Fisioterapia. Recife, PE, Brasil; V Brasil Secretaria Estadual de Saúde do Rio Grande do Norte, Natal, RN, Brasil; VI Brasil Instituto Federal de Educação, Ciência e Tecnologia do Rio de Janeiro, Rio de janeiro, RJ, Brasil

**Keywords:** Centros de Reabilitação, organização & administração, Pessoas com Deficiência, reabilitação, Gestão de Recursos da Equipe de Assistência à Saúde, Avaliação de Processos e Resultados em Cuidados de Saúde, Administração de Recursos Humanos

## Abstract

**OBJETIVO:**

Construir e validar um modelo lógico para a atenção nos Centros Especializados em Reabilitação (CER) a partir da análise do processo de trabalho e de questões organizativas de centros do Rio Grande do Norte.

**MÉTODOS:**

Estudo metodológico desenvolvido em três etapas: 1) estudo documental de legislações e portarias relacionadas ao serviço de saúde e à Rede de Cuidados à Pessoa com Deficiência (RCPD); 2) realização de grupos focais, com estudo censitário dos CER do Rio Grande do Norte, para compreender e avaliar o cotidiano do serviço; e 3) sistematização das informações coletadas e, por fim, proposição e validação do modelo lógico avaliativo.

**RESULTADOS:**

O modelo englobou cinco categorias centrais do processo de trabalho e organizacional: “demandas”, “recursos” (insumos, financeiros e força de trabalho), “processos”, “produtos e resultados” e “missão, valores e fatores externos”.

**CONCLUSÃO:**

O modelo lógico construído foi adequado para representação gráfica do processo de trabalho e questões organizativas dos CER. Evidenciou-se que o funcionamento dos serviços está alinhado com as normativas. Contudo, ainda há lacunas organizacionais que precisam ser abordadas a fim de melhorar a resolutividade do serviço e a articulação com outros pontos da rede.

## INTRODUÇÃO

Em virtude do maior número de pessoas vivendo com deficiência, do aumento da incidência de doenças crônicas e do envelhecimento populacional, o tema da reabilitação tem ganhado destaque no Brasil e no mundo^1^. A agenda “Chamada para a Ação: Reabilitação 2030”, proposta pela Organização Mundial da Saúde (OMS), defende o acesso universal à reabilitação e convoca as nações a refletirem sobre como definem políticas públicas, estruturam seus serviços de assistência e sistemas de informação^1,2^. No Brasil, a escassez de bibliografia e instrutivos relacionados aos serviços de reabilitação, ofertados por Centros Especializados em Reabilitação (CER), denota a urgência e pertinência de estudos que avaliem essa área da assistência.

De acordo com a OMS, uma em cada sete pessoas no mundo vive com deficiência^3^. No Brasil, os dados são defasados, já que o panorama mais atual é fornecido pelo Censo de 2010. De acordo com esse levantamento, 23,9% da população brasileira tem ao menos um tipo de deficiência, e a prevalência aumenta com a idade^4^.

Criados pela Portaria nº 793/2012, que instituiu a Rede de Cuidados à Pessoa com Deficiência (RCPD), os CER são serviços de saúde que ofertam atenção especializada em reabilitação para pessoas com deficiência em todo o território nacional^5^. Esses centros são referências para a RCPD e para as políticas públicas brasileiras de atenção à pessoa com deficiência^6^.

Os CER podem assistir pessoas com deficiência física, auditiva, visual, intelectual e múltiplas deficiências. De acordo com o perfil epidemiológico do território, esses centros podem ser habilitados em três formatos: CER II, composto por duas modalidades de reabilitação; CER III, composto por três modalidades de reabilitação; e CER IV, composto por quatro modalidades de reabilitação (física, visual, auditiva e intelectual), além da oficina ortopédica^5^.

Dentre as vertentes de avaliação em saúde, a análise da implementação de programas ou serviços por meio de modelos lógicos vem ganhando destaque^7^. O modelo lógico é uma representação gráfica que evidencia as diversas relações possíveis entre as atividades previstas e os resultados esperados^8^, facilitando o planejamento das ações do serviço e a comunicação entre os sujeitos envolvidos na sua condução^7^. Nessa perspectiva, o modelo lógico é uma ferramenta útil para desenvolver, implementar e avaliar processos complexos, pois permite uma visão geral, capaz de identificar atividades e desfechos-chave^8,9^.

Assim, considerando que a construção desses modelos para serviços de reabilitação fornece uma visão geral dos processos complexos envolvidos, gerando informações relevantes para a tomada de decisão, o presente trabalho objetiva construir e validar um modelo lógico para a atenção especializada nos CER. Para isso, a pesquisa parte da análise do processo de trabalho e de questões organizativas no estado do Rio Grande do Norte.

## MÉTODOS

Trata-se de estudo metodológico de caráter descritivo, com abordagem qualitativa, desenvolvido em três etapas: análise documental, grupos focais e, por fim, construção e validação de um modelo lógico representativo do funcionamento dos CER^8,10^. O protocolo de pesquisa foi aprovado pelo Comitê de Ética em Pesquisa da Faculdade de Ciências da Saúde do Trairi sob o número CAAE 07082819.3.0000.5568. O estudo foi conduzido em consonância com a Declaração de Helsinque, e os participantes assinaram termo de consentimento livre e esclarecido, bem como autorizaram a gravação de voz.

A primeira etapa consistiu em estudo de portarias, legislações e documentos relacionados à RCPD e aos CER. A análise documental permitiu entender as diretrizes e normatizações aplicáveis à rede de saúde e ao serviço a ser avaliado, bem como a identificação prévia de conceitos-chave úteis à construção do modelo lógico^10^.

Na segunda etapa, foram organizados grupos focais, com profissionais e gestores, a fim de compreender processos cotidianos do serviço^11^. Para otimizar as questões logísticas e ao mesmo tempo manter a representatividade amostral^12^, optou-se por uma amostragem censitária, com todos os CER de uma RCPD estadual. Por conveniência e inserção dos pesquisadores, foram escolhidos nove CER do Rio Grande do Norte que, juntos, representam as oito regiões de saúde do estado. Cada serviço foi representado por um gestor e um profissional assistencial, formando assim uma composição paritária. Participaram do grupo focal profissionais de diferentes classes: assistência social, enfermagem, fisioterapia, fonoaudiologia, medicina, odontologia, psicologia e terapia ocupacional.

As sessões dos grupos focais ocorreram com periodicidade mensal, como parte do Fórum de Gestão da RCPD. Inicialmente, apenas o gestor de cada serviço havia sido convidado. Contudo, a pedido dos próprios serviços, foram incluídos profissionais que já participavam regularmente dos fóruns de gestão da rede. Além dos dois representantes dos serviços (o gestor e um profissional da assistência), cada grupo focal contou com a participação de dois profissionais de saúde, externos à RCPD, com experiência em gestão e avaliação em saúde.

A condução do grupo ficou a cargo de equipe composta por um moderador e dois observadores que registraram aspectos relacionados à linguagem não verbal dos participantes (identificados com crachás) e a sequência e o conteúdo das falas^11^. Ao longo dos grupos focais, buscou-se apreender na fala dos participantes: demandas do serviço, causas e consequências da demanda, objetivos do serviço, público-alvo, recursos, ações, produtos, resultados e fatores que interferem no desempenho do serviço^10^.

Ao todo foram realizados cinco encontros, entre os meses de março e julho de 2019, cada um com duração média de 90 minutos. O áudio foi captado por dois gravadores digitais, posicionados nos extremos das mesas. As falas foram posteriormente transcritas e as informações categorizadas pela análise temática de conteúdo^11^, com base no arcabouço teórico da atenção à pessoa com deficiência, dos cuidados em rede e da avaliação em saúde.

O modelo lógico foi elaborado pelos pesquisadores com base nas informações coletadas nas etapas anteriores, mas sem a presença de profissionais ligados ao CER^10^. Para apresentação gráfica, construiu-se uma versão adaptada, baseada em proposta de Tamaki et al.^13^. Foram acrescentados ao modelo os domínios “missão”, “valores” e “fatores externos”.

O modelo foi submetido a validação de conteúdo no último grupo focal com os representantes dos CER^10^. Nesse encontro, os moderadores do grupo apresentaram o modelo lógico por meio de projeção multimídia e esclareceram os participantes sobre os domínios contemplados: demandas, recursos (insumos, recursos financeiros e força de trabalho), processos, produtos, resultados e fatores externos. Em seguida, a equipe checou itens e ideias contidas no modelo, domínio por domínio. Os representantes dos serviços de saúde opinaram livremente e puderam sugerir exclusões, inclusões ou modificações na alocação dos itens. A versão final, para ser considerada validada, deveria ser aprovada por consenso entre os participantes.

## RESULTADOS

Criados em 2012 como componente principal da atenção especializada à pessoa com deficiência, os CER são responsáveis por ações de reabilitação e habilitação. Esses centros coexistem com serviços de modalidade única de reabilitação (filantrópicos em sua maioria), buscando suprir o vazio assistencial e promover uma atenção integral à pessoa com deficiência, de forma articulada com outros pontos da RCPD^14^.

Os CER fazem parte de uma política pública de saúde que compreende agentes, estruturas, processos e objetivos de cuidado predeterminados^15^. Essa política, orientada à funcionalidade e ao modelo biopsicossocial, visa ofertar serviços de reabilitação consonantes com os princípios e diretrizes da RCPD.

Essa política ainda está sendo implementada, e pode-se dizer que a missão dos CER ainda é pouco compreendida tanto por parte dos profissionais como dos usuários do sistema de saúde. No cotidiano dos serviços, há dificuldades patentes em relação a cobertura, acesso, qualidade da assistência, planejamento terapêutico, recursos humanos e materiais.

Considerando que certos arranjos organizativos do trabalho podem impedir a integralidade do cuidado^16^, buscou-se desenvolver um modelo lógico de intervenção que respondesse às seguintes questões: como funcionam os CER? Quais são os elementos da política brasileira de cuidado à pessoa com deficiência e como eles se relacionam? Os objetivos propostos nessa política caminham para a inclusão? Quais os motivos que levam a política de reabilitação a seguir ou não as diretrizes da RCPD?

O modelo lógico elenca os principais tópicos dos processos organizativos e de trabalho dos CER, dividindo-os em seis domínios (Figura 1). Vale destacar que o modelo construído a partir de arcabouço teórico e das discussões nos grupos focais foi aprovado por consenso, sem modificações. O Quadro 1 sintetiza as temáticas e elementos representativos dos domínios contemplados no modelo.

**Figura:** Modelo lógico para os Centros Especializados em Reabilitação.

**Quadro 1:** Detalhamento dos elementos constituintes do modelo.

Domínio e definição^a^	Principais comentários
**Demandas** Os sujeitos e seus problemas.	As deficiências podem ser adquiridas ao longo da vida, e todo sujeito é um potencial usuário dos Centros Especializados em Reabilitação (CER). As necessidades da família e cuidadores devem ser incluídas como demandas do serviço, o que amplia o público-alvo das ações do CER.
**Recursos** Recursos podem ser financeiros, força de trabalho ou insumos.	Financiamento tripartite pelo Bloco de Custeio de Média e Alta Complexidade (MAC). Esse tipo de financiamento pode ser um entrave substancial para a alocação dos recursos no serviço destinatário, que não é garantida por esse rito administrativo. Incompatibilidade dos valores oferecidos pelo Sistema de Gerenciamento da Tabela de Procedimentos, Medicamentos e Órteses, Próteses e Meios Auxiliares de Locomoção (Sigtap) e os valores de mercado. Os recursos humanos correspondem à equipe multiprofissional, formada por diferentes categorias da saúde, além da equipe de apoio e administrativa. Percebe-se a dificuldade dos profissionais em atuar de forma interdisciplinar, ofertando o cuidado integralmente, com foco na funcionalidade humana. Essa dificuldade parece se dever à formatação do trabalho e à própria formação dos profissionais, já que os CER são um serviço recente.
**Processos** Ações e ferramentas para alcançar os produtos e resultados esperados.	Avaliação global, com classificação da funcionalidade humana por meio da Classificação Internacional de Funcionalidade, Incapacidade e Saúde – embora essa classificação seja uma realidade em poucos serviços. Consultas compartilhadas, projeto terapêutico singular, ações de educação em saúde, grupos terapêuticos e alta qualificada. Ações assistenciais, matriciamento, alimentação de sistemas de informação e articulação com outros serviços.
**Produtos** Materialização do esforço dispendido nos processos.	Oferecer habilitação e reabilitação às pessoas com deficiência, contribuindo para que esses sujeitos desempenhem suas funções e atividades diárias com independência, além de garantir um apoio familiar capaz de garantir a convivência harmônica entre a pessoa com deficiência e seu núcleo familiar.
**Resultados** Repercussão e impacto a médio e longo prazo.	Promover a inclusão social da pessoa com deficiência, seja no contexto escolar ou profissional, melhorando sua qualidade de vida e garantindo a participação social e o exercício da cidadania.
**Fatores externos** Fatores que fogem à competência da equipe de profissionais e podem interferir no desempenho do serviço.	Fatores que impactam positivamente: o Fórum de Gestão da Rede de Cuidados à Pessoa com Deficiência, espaço consultivo e deliberativo sobre os serviços ofertados, que possibilita a troca de experiências e a ajuda mútua entre os serviços, favorecendo uma assistência de qualidade; parcerias entre os CER e instituições de ensino técnico e superior para qualificar os recursos humanos e permitir que profissionais em formação vivenciem o serviço na prática. Fatores que impactam negativamente: inflexibilidade das portarias que regulamentam o CER; dificuldade com a alocação dos recursos, destinados exclusivamente para custeio; falta de instrumentos para avaliar a qualidade dos serviços e das ações; dificuldade na realização de contrarreferência e referência; forma de habilitação dos serviços por tipos de deficiência, desconsiderando a integralidade do cuidado e limitando o processo de reabilitação do indivíduo.

^a^ As definições dos domínios do modelo lógico foram elaboradas de acordo com o modelo teórico proposto por Tamaki et al.^13^

Durante os grupos focais, buscou-se identificar dificuldades e problemas reais enfrentados cotidianamente pelos gestores e profissionais, assim como causas, consequências e soluções desses problemas. A síntese dessas informações encontra-se no Quadro 2.

**Quadro 2:** Síntese dos problemas e desafios vivenciados na rotina do serviço a partir da ótica de profissionais e gestores.

Problemas	Causas	Consequências	Estratégia de enfrentamento
Baixa visibilidade das pessoas com deficiência.	Estigma social, falta de informações sobre as deficiências.	Exclusão social e isolamento. Subvalorização dos serviços e da atenção à pessoa com deficiência (PcD).	Ações educativas sobre os direitos da PcD e a importância dos Centros Especializados em Reabilitação (CER).
Dificuldade em aplicar o modelo biopsicossocial na avaliação global, na definição de metas terapêuticas e na alta qualificada.	Déficit na comunicação entre os profissionais e entre estes e o paciente/família. Práticas biologicistas e avaliação fragmentada por categoria profissional. Baixa ou nenhuma adesão à Classificação Internacional de Funcionalidade, Incapacidade e Saúde (CIF) ou instrumentos nela baseados. Resistência da família/cuidadores em aceitar a alta, por receios relativos à continuidade do cuidado em ambiente domiciliar ou do Benefício de Prestação Continuada (BPC). Baixa capacidade dos profissionais para gerir o cuidado e inexistência de avaliação da qualidade da atenção.	Fragmentação da assistência e projetos terapêuticos baseados no modelo biomédico, com práticas unilaterais, estabelecidas sem diálogo. Dificuldade para definir as prioridades terapêuticas e alocar profissionais no caso. Falta de autonomia do paciente e da família na gestão do cuidado. Baixa resolutividade das ações, com permanência dos pacientes por tempo superior ao necessário e aumento da demanda reprimida.	Reuniões periódicas entre profissionais e familiares para desenvolver um projeto terapêutico singular. Avaliação global baseada na CIF, com presença de toda a equipe de profissionais. Articulação com outros pontos da rede para continuidade do cuidado. Construção e revisão de protocolos para orientar a atenção à saúde disponibilizada pelo CER. Desenvolvimento de indicadores de avaliação e acompanhamento da qualidade da atenção oferecida nos CER.
Dificuldade na articulação do CER com outros serviços da Rede de Cuidados à Pessoa com Deficiência (RCPD), com outras redes de atenção e com outros setores.	Pouco reconhecimento e valorização das CER dentro das redes de atenção. Falhas na comunicação entre o CER e outros serviços integrantes da RCPD e outras redes de atenção. Baixa qualificação dos profissionais de saúde e de outros setores para atenção à pessoa com deficiência e promoção da inclusão. Dificuldades logísticas como falta de transporte sanitário e compatibilização de agendas.	Pouca ou nenhuma articulação intersetorial. Descontinuidade do cuidado e inserção social da PcD. Agravamento do distanciamento entre atenção especializada, outros pontos da rede e participação social da PcD.	Reuniões periódicas com a presença de representantes de redes de atenção e setores com outros níveis de densidade tecnológica. Desenvolvimento de fluxogramas para ilustrar a relação entre os serviços e protocolos para encaminhamento.
Inflexibilidade das portarias do Ministério da Saúde e da alocação de recursos.	Desconsideração das singularidades e realidades locais. Comunicação deficitária ou inexistente entre o CER e o Ministério da Saúde. Incompatibilidade dos valores na Tabela SUS com o custo real do produto/serviço. Recurso mensal da forma de custeio. Má gestão dos recursos. Recursos conjuntos para CER e outros serviços. Burocratização para implantação de novos CER e desinteresse dos gestores para fazê-lo.	Fragmentação do cuidado pelo tipo de deficiência, levando o paciente a percorrer vários pontos da rede em casos de deficiências múltiplas ou necessidades diferentes da habilitação do CER mais próximo. Necessidade de realocar outros recursos para suprir as demandas financeiras ou desvio do recurso do CER repassado pela MAC. Dificuldade de avanço tecnológico do serviço. Demanda elevada para o serviço e inadequação da assistência ofertada para a realidade do território.	Publicização de estudos sobre os CER para problematizar a necessidade de mudanças nos instrumentos normativos. Escuta dos atores envolvidos no CER para elaborar diretrizes aplicáveis ao serviço e à atenção à PcD. Reconsideração das habilitações por tipo de deficiência e criação de sistemas de regulação para acesso a atendimentos especializados e insumos terapêuticos como órteses e próteses.
Dificuldades relacionadas aos sistemas de informação	Utilização das informações contidas nos sistemas para repasse financeiro. Prontuários eletrônicos não unificados entre atenção básica e CER. Sistemas de informação baseados no diagnóstico e fatores biológicos da deficiência, desconsiderando a influência do contexto na produção das deficiências.	Sistemas de informação não refletem a qualidade dos serviços, que acabam recebendo má avaliação. Fragmentação das informações de saúde do indivíduo, dificultando o acesso a dados pelas equipes de reabilitação e Estratégia Saúde da Família, o que compromete a integralidade do cuidado. Ausência de sistemas de informação que permitam gerar dados sobre funcionalidade, limitação às atividades, restrição à participação e fatores contextuais.	Estímulo a uma cultura organizacional que promova valores de cooperação no serviço, planejamento de ações e avaliação dessas ações. Ampliação do Prontuário Eletrônico do Cidadão no e-SUS para incluir informações sobre o CER, viabilizando uma análise geral do indivíduo através do prontuário. Utilização de sistemas de informação com dados baseados na CIF.
Dificuldades relacionadas à permanência e qualificação dos profissionais, estrutura física adequada e terapias alternativas.	Profissionais vinculados ao serviço por contrato. Baixa afinidade de profissionais com o modelo assistencial do CER. Jornada dupla de trabalho, com atividades em outros pontos de atenção. Recursos financeiros insuficientes para educação continuada da equipe.	Alta rotatividade da equipe assistencial. Dificuldade de estabelecer e manter o vínculo profissional-paciente. Defasagem na qualificação da equipe. Tratamento curativo, baseado no modelo biomédico.	Realização de concursos públicos e instituição de avaliação de desempenho para estimular a permanência de profissionais aptos ao serviço. Avaliação periódica dos CER para verificar questões relacionadas a estrutura, processos e resultados obtidos.

## DISCUSSÃO

O modelo lógico é uma ferramenta importante para a tomada de decisão em saúde, pois permite que a equipe tenha uma visão geral dos processos desenvolvidos, identificando pontos fortes e entraves à obtenção dos desfechos esperados (sejam produtos ou resultados)^8^. O modelo aqui proposto, baseado em estudo de caso de serviços que compõem a RCPD no Rio Grande do Norte, foi validado por profissionais e está em consonância com diretrizes internacionais para gestão do cuidado e reabilitação^17^ e com normatizações dos CER no Brasil^5,21,22^.

Em comparação com outros países, a definição de políticas e programas direcionados à pessoa com deficiência ainda é recente no Brasil. São marcos desse processo a Política Nacional de Saúde da Pessoa Portadora de Deficiência^21^ e a Portaria nº 793/2012^5^. Essa portaria estabelece como atribuições dos CER: fornecer diagnóstico e tratamento oportuno em tempo adequado; realizar concessão, adaptação e manutenção de tecnologias assistivas; atuar de forma articulada e como matriz sobre a temática da deficiência com outros pontos da rede; e prevenir deficiências e agravos adicionais^5^.

Os serviços oferecidos pelos CER fortalecem os direitos conquistados pelas pessoas com deficiência ao longo dos anos^21^. Esses centros, como mostra o modelo lógico aqui apresentado, estão em consonância com as recomendações da OMS para fortalecer a reabilitação nos sistemas de saúde. Os CER garantem o acesso da comunidade a serviços de reabilitação, disponibilizando uma força multiprofissional e alocando recursos financeiros para oferecer dispositivos e tecnologias assistivas a todos que necessitem, assim como treinamento adequado para seu uso seguro e eficaz^22^. Contudo, destaca-se que ainda há necessidade de maior integração entre os diferentes níveis de atenção e serviços, tendo em conta a dinamicidade das limitações e necessidades individuais que, além da saúde, podem demandar ações de educação, trabalho, lazer e cultura, entre outras^22,24,25^.

Os CER são serviços recentes na lógica do SUS^23^, e nos grupos focais ficou evidente que algumas ações ainda não se consolidaram. Como exemplo, podem-se citar dificuldades relativas à avaliação global e à utilização da Classificação Internacional de Funcionalidade, Incapacidade e Saúde (CIF) no planejamento terapêutico. Os profissionais também apontam inexistência de assistência social, de indicadores de qualidade e de alta qualificada, com encaminhamento oportuno para outros serviços.

A CIF deve ser encarada como um dos pilares da filosofia do cuidado à pessoa com deficiência^5,22^. Sua utilização no planejamento terapêutico permite produzir dados e alimentar sistemas de informação de forma padronizada. Com a CIF também é possível otimizar a alocação de profissionais e identificar as intervenções necessárias para cada caso, direcionando o processo terapêutico à funcionalidade humana, deixando de considerar apenas a função biológica que se encontra prejudicada temporária ou permanentemente^1,20,22,26^. Assim, o uso da CIF desloca o planejamento terapêutico de uma perspectiva biomédica para um modelo biopsicossocial e holístico, contribuindo para o melhor desenho dos serviços e para o desenvolvimento de políticas públicas voltadas às pessoas com deficiência^2,20,26^.

Quanto à alta, os participantes do grupo focal a consideraram um momento problemático na rotina dos CER. Para reverter esse cenário, é preciso que os cuidados à pessoa com deficiência articulem diferentes serviços e níveis de atenção^27^. O CER precisa ser visto como ponto de atenção especializada, e não como único serviço direcionado à pessoa com deficiência. Sua atuação deve ser matricial^23^, coordenando ações assistenciais e conscientizando a sociedade e outros serviços sobre seu papel social, de modo a facilitar a compreensão dos envolvidos no cuidado e otimizar o acesso e os encaminhamentos. Quando isso não ocorre, os canais de comunicação entre profissionais e serviços se tornam ineficientes, e a incompreensão do papel do CER dentro da rede pode gerar o subaproveitamento de suas ações^27,28^.

A falta de uma cultura de avaliação é uma constante no sistema de saúde brasileiro^29^. À inexistência de avaliação interna da qualidade dos processos e serviços prestados se soma a insuficiência de profissionais que atuam na reabilitação. A distribuição desigual dos profissionais e serviços de saúde agravam a situação da assistência às pessoas com deficiência, que tem apresentado qualidade abaixo da ideal^29,30^.

Dentre os fatores externos que interferem no desempenho do serviço, a crítica à forma de habilitação dos CER é uma questão central. Quando se habilitam serviços de referência apenas para alguns tipos de deficiência (ainda que com base em diagnóstico territorial), a atenção se fragmenta, desconsiderando as múltiplas deficiências. As necessidades de saúde das pessoas não são padronizadas e podem variar dentro de um mesmo território de saúde, de modo que seria necessário flexibilizar as regulamentações, para que cada território adeque o serviço a sua realidade e ao mesmo tempo garanta acesso aos serviços de reabilitação de modo oportuno, sem que o paciente precise percorrer grandes distâncias^22,29^.

Além disso, observa-se concentração dos serviços de atenção especializada em capitais, metrópoles e polos regionais^23^. Essa concentração acaba obrigando as pessoas com deficiência a se deslocar por grandes distâncias em busca de atendimento, o que vai contra a recomendação da OMS de que serviços de reabilitação estejam disponíveis o mais próximo possível das comunidades, inclusive das comunidades rurais^23,26,30^.

Embora tenha se baseado em estudo realizado em apenas um estado da federação brasileira, o modelo aqui apresentado demonstrou-se alinhado a teorias e normatizações nacionais e internacionais. Seu uso em serviços de outras regiões, entretanto, deve considerar o contexto de cada serviço. Além disso, para estudos futuros, recomenda-se que, além da percepção de gestores e profissionais, também a percepção dos usuários seja contemplada. Essa abordagem pode promover o protagonismo da pessoa com deficiência, como preconiza a Convenção Internacional sobre os Direitos da Pessoa com Deficiência^23^.

Como limitação do estudo, apontam-se ausências de profissionais assistenciais em alguns grupos focais, em virtude de choques de agenda e outros compromissos. Contudo, acreditamos que essa limitação não repercutiu de forma significativa nos resultados do estudo, uma vez que o modelo foi validado sem correções. Ademais, todos os participantes foram indicados pelos próprios serviços e tinham vivência em CER.

Por meio da comparação do modelo validado – que representa o funcionamento ideal dos serviços – com a realidade local, os profissionais e gestores poderão avaliar os serviços em que atuam, identificando potencialidades e desafios. Dessa forma, o modelo lógico pode funcionar como instrumento de gestão para conhecer a implementação do serviço, auxiliar a tomada de decisões em saúde e, em última análise, favorecer o planejamento de ações assistenciais para pessoas com deficiência.

Além disso, a partir da identificação dos pontos cruciais do serviço, o modelo pode ser útil para criar indicadores de desempenho aplicáveis ao CER, contribuindo para o desenvolvimento de uma cultura avaliativa nos serviços, seja por processos internos ou externos de avaliação de qualidade.

O modelo lógico e a identificação dos problemas associados ao funcionamento dos CER demonstram que a política de reabilitação para pessoas com deficiência ainda está em processo de implementação no Brasil. Embora tal política tenha um desenho alinhado às diretrizes de cuidado mais atuais, na prática há barreiras no acesso, no fluxo dos pacientes e nas ações ofertadas. É preciso, portanto, reorientar o serviço a fim de que ele cumpra as diretrizes do cuidado integral, com foco na funcionalidade humana, e atinja os resultados esperados: melhorar a qualidade de vida e promover a inclusão social da pessoa com deficiência.
